# Functional ultrasound imaging reveals microvascular rarefaction, decreased cerebral blood flow, and impaired neurovascular coupling in a mouse model of paclitaxel-induced chemobrain

**DOI:** 10.1007/s11357-025-01624-7

**Published:** 2025-03-25

**Authors:** Siva Sai Chandragiri, Adam Nyul-Toth, Sharon Negri, Roland Patai, Rafal Gulej, Boglarka Csik, Santny Shanmugarama, Kiana Vali Kordestan, Mark Nagykaldi, Peter Mukli, Anna Ungvari, Andriy Yabluchanskiy, Zoltan Ungvari, Stefano Tarantini, Anna Csiszar

**Affiliations:** 1 Vascular Cognitive Impairment, Neurodegeneration and Healthy Brain Aging Program, Department of Neurosurgery, University of Oklahoma Health Sciences Center, Oklahoma City, OK USA; 2 Oklahoma Center for Geroscience and Healthy Brain Aging, University of Oklahoma Health Sciences Center, Oklahoma City, OK USA; 3 International Training Program in Geroscience, Doctoral College, Health Sciences Division/Institute of Preventive Medicine and Public Health, Semmelweis University, Budapest, Hungary; 4 Institute of Preventive Medicine and Public Health, Semmelweis University, Budapest, Hungary; 5 Department of Health Promotion Sciences, College of Public Health, University of Oklahoma Health Sciences Center, Oklahoma City, OK USA; 6 The Peggy and Charles Stephenson Cancer Center, University of Oklahoma Health Sciences Center, Oklahoma City, OK USA; 7 International Training Program in Geroscience, Doctoral College/Institute of Translational Medicine, Semmelweis University, Budapest, Hungary

**Keywords:** Accelerated aging, Taxol, Brain microcirculation, Cerebral circulation, Capillary density, Endothelial dysfunction, Senescence, VCID, Vascular cognitive impairment, Endothelial cell

## Abstract

Chemotherapy-induced cognitive impairment (CICI), often referred to as “chemobrain,” significantly affects the quality of life in cancer survivors. Although traditionally attributed to neuronal toxicity, emerging evidence suggests a key role of cerebrovascular dysfunction in its pathogenesis. We hypothesized that paclitaxel (PTX, Taxol) treatment induces long-term cerebrovascular dysfunction, including microvascular rarefaction, impaired neurovascular coupling (NVC), and altered cerebral blood flow (CBF), which contribute to CICI. Using a clinically relevant PTX treatment regimen in non-tumor-bearing mice, we evaluated the long-term effects of PTX on cerebrovascular health. Ultrasound localization microscopy (ULM) and functional ultrasound imaging (fUS) were employed to assess microvascular density, CBF, and NVC. PTX treatment resulted in a significant reduction in microvascular density in the cerebral cortex and hippocampus, key regions involved in cognitive function. PTX significantly reduced blood velocity in the middle cerebral artery. Moreover, PTX impaired NVC responses, as evidenced by a diminished CBF increase in response to whisker stimulation, indicative of impaired reactive hyperemia. In conclusion, these findings demonstrate that PTX induces long-lasting cerebrovascular dysfunction, including microvascular rarefaction, impaired NVC, and altered CBF dynamics, which likely contribute to CICI. This study underscores the critical role of cerebrovascular health in cognitive function and highlights the potential of targeting cerebrovascular pathways as a therapeutic approach for mitigating chemotherapy-induced cognitive deficits.

## Introduction

Chemotherapy-induced cognitive impairment (CICI), commonly referred to as “chemobrain,” is a significant side effect of cancer treatment, affecting an estimated 30–50% of patients treated with chemotherapeutic agents, including paclitaxel (PTX) [1–3]. CICI manifests as deficits in memory, attention, and executive function, often persisting long after treatment and severely impairing quality of life and overall treatment outcomes [1–3]. Despite considerable advancements in cancer therapy, effective strategies to prevent or mitigate CICI remain elusive, underscoring the critical need to elucidate its underlying mechanisms [4].

PTX, a widely used chemotherapeutic agent, is a cornerstone in the treatment of breast, ovarian, lung, and other cancers. Its primary mechanism of action involves stabilizing microtubules and disrupting cellular mitosis, effectively targeting rapidly dividing tumor cells. However, PTX is also associated with numerous off-target effects, including neurotoxicity [5–10] and CICI [1–3,11–13]. PTX does not cross the blood–brain barrier (BBB) in significant amounts due to its high molecular weight and poor lipophilicity and the presence of P-glycoprotein efflux transporters at the BBB [14]. Emerging evidence indicates that the cerebral microvascular endothelium serves as the first line of contact with circulating chemotherapeutics, and that PTX-induced accelerated aging-like endothelial effects contribute to microvascular alterations that may underlie CICI [15]. Recent preclinical studies have shown that PTX adversely affects brain microvascular endothelial cells, inducing cellular senescence and leading to complex microvascular dysfunction, including persistent BBB disruption [15]. Despite these advances, critical gaps remain in understanding how PTX impacts cerebral blood flow regulation, a key factor in maintaining neural homeostasis and cognitive function.

In this study, we hypothesize that previously demonstrated PTX treatment-induced endothelial senescence leads to cerebromicrovascular rarefaction, impaired neurovascular coupling (NVC) responses, and reduced cerebral blood flow (CBF) [15]. These microvascular disruptions are proposed to contribute to the neuronal dysfunction underlying CICI. To test these hypotheses, we treated non-tumor-bearing mice with a clinically relevant PTX regimen to isolate the direct vascular effects of the drug, minimizing potential confounding factors associated with tumor biology. Functional ultrasound (fUS) imaging, an advanced modality offering superior spatiotemporal resolution, was used to evaluate microvascular density, NVC responses, and CBF in PTX-treated and control mice. Unlike traditional imaging techniques such as fMRI or two-photon microscopy, fUS enables detailed longitudinal assessment of cerebral vasculature across the entire brain [16,17]. This powerful approach allowed us to capture dynamic changes in brain perfusion and vascular structure, providing novel insights into the microvascular contributions to PTX-induced cognitive impairment.

## Materials and methods

### Experimental animals and study design

**Fig. 1:**
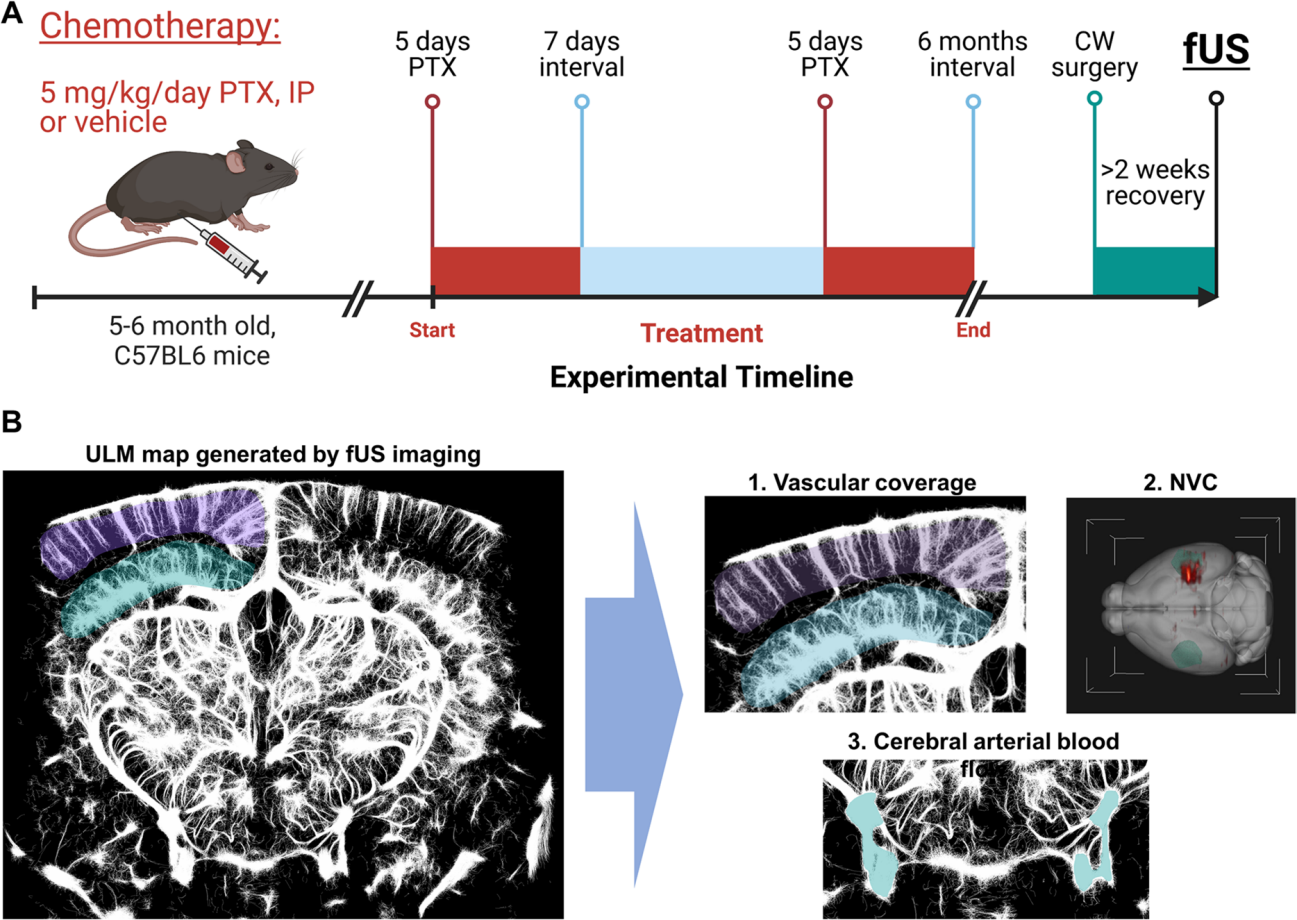
Experimental timeline and imaging modalities for assessing chemotherapy-induced cognitive impairment (CICI). **A** Schematic representation of the experimental timeline for the C57BL/6 mouse model of CICI. Mice were treated with paclitaxel (PTX; 5 mg/kg/day, intraperitoneally) or vehicle in two 5-day cycles separated by a 7-day interval. Functional ultrasound (fUS) imaging was performed 6 months after the final treatment, following cranial window (CW) surgery and a 2-week recovery period. **B** Imaging capabilities of the ICONEUS fUS system for assessing cerebrovascular health. Ultrasound localization microscopy (ULM) maps provide high-resolution visualization of vascular coverage (1), neurovascular coupling (NVC) responses (2), and cerebral arterial blood flow dynamics (3)

C57BL/6 mice (6 months old) were obtained from Jackson Laboratories (Bar Harbor, ME) and housed three per cage in the specific pathogen-free animal facility at the University of Oklahoma Health Sciences Center (OUHSC). Mice were maintained under a 12-h light/dark cycle with ad libitum access to standard rodent chow and water. One week before chemotherapy treatment, mice were transferred to a conventional facility under similar housing conditions. PTX was administered intraperitoneally (5 mg/kg/day) in two cycles (5 days/cycle), separated by a 1-week interval. Vehicle-treated mice (DMSO + saline) served as controls. Cerebrovascular assessments were conducted 6 months after treatment, following cranial window surgery and recovery (Fig. 1A). All animal protocols were approved by the Institutional Animal Care and Use Committee of OUHSC.

### Cranial window implantation

A chronic polymethylpentene plastic (TPX) window was surgically implanted as described by Nyul-Toth et al. [18]. The procedure was performed 2–3 weeks before imaging to ensure full recovery and stable conditions for subsequent experiments. Mice were anesthetized with isoflurane (3–4% for induction and 2–3% for maintenance) delivered at a flow rate of 0.6–0.8 L/min. Once fully anesthetized, confirmed by the absence of paw and tail reflexes, the mice were positioned on a stereotactic stage under a stereomicroscope, and ophthalmic ointment was applied to protect their eyes. The surgical site was prepared with povidone-iodine and 70% ethanol to ensure sterility. A 10 × 15 mm incision was made to expose the skull, which was then scraped clean with a scalpel. To minimize pain and discomfort, lidocaine (2%) was applied to the skull before performing the craniotomy. The skull was gradually thinned using intermittent drilling to avoid heat-induced tissue damage, and the bone was carefully removed using fine forceps. Bleeding was managed using saline-soaked gelatin sponges. A custom-curved TPX™ film (4 × 8 mm, 0.125-mm thickness) was sterilized in 70% ethanol and placed over the craniectomy site, ensuring a snug fit that avoided undue pressure on the underlying tissue. The window was secured with biocompatible adhesive and Chromic 5–0 sutures, creating a stable and contamination-resistant interface. Postoperative care included administration of extended-release buprenorphine (1 mg/kg) for pain management and daily antibiotics (Baytril, 10 mg/kg) for 4 days. The mice were monitored continuously until full recovery, with food and water accessible on the cage floor to facilitate recuperation. Postoperative monitoring continued for 2 weeks to ensure optimal recovery before imaging.

### Imaging of the brain vasculature with ICONEUS ONE fUS

Ultrasound localization microscopy (ULM) is a super-resolution imaging technique that surpasses the diffraction limit of conventional ultrasound by detecting and tracking individual microbubbles (MBs) injected intravenously. This innovative method enables high-resolution visualization of microvascular structures and blood flow dynamics in vivo. ULM complements functional ultrasound (fUS) by providing unprecedented detail in imaging microvasculature, achieving two orders of magnitude improvement in vascular resolution. This allows precise mapping of deep brain vasculature and blood flow dynamics [19–21]. ULM has been successfully applied to quantify neurovascular activity across large brain regions at a microscopic scale [22], making it a powerful tool for studying both physiological and pathological cerebrovascular processes, including those induced by chemotherapy. Together, fUS and ULM provide complementary insights into cerebrovascular dynamics, offering critical tools for understanding the vascular contributions to cognitive dysfunction and identifying potential therapeutic strategies [23,24].

Using the transparent TPX window, high-resolution imaging (2 µm/pixel) was conducted from the brain’s surface to its base, providing detailed data on microvascular architecture and flow dynamics, essential for analyzing vascular function in vivo. For imaging, mice in each group were anesthetized with isoflurane (3% for induction and 1% for maintenance) and intubated with a 20Gx1 catheter (Exel, CA, USA). Mechanical ventilation was performed using a MousVent G500 system (Kent Scientific Co, Torrington, CT), maintaining body temperature at 37 °C with a thermostatic heating pad. End-tidal CO₂ levels were regulated between 3.2 and 3.7% to ensure physiological blood gas values. The mice were immobilized on a stereotaxic frame with an anesthesia mask (51,625 W, Stoelting Co, Wood Dale, IL, USA) connected to a ventilator (SomnoSuite® Low-Flow Anesthesia System, Kent Scientific Corporation) to minimize respiratory-induced hemodynamic fluctuations.

The surgical site was prepared by shaving the head with a depilatory cream (Nair hair removal body cream, Aloe) and cleaning it with ethanol. The cranial window was exposed by making a central incision and ensuring no tissue or blood contamination. An ultrasonic probe (IcoPrime-4D MultiArray 15 MHz, ICONEUS, France) from the ICONEUS One fUS device was positioned above the cranial window and submerged in ultrasound gel (Gel de contact, Drexco Medical, Crosne, France).

ULM relies on detecting and tracking individual MBs injected intravenously. For this study, 50 µL of sterile MB suspension (DEFINITY, Lantheus, Billerica, MA, USA) was prepared by agitating the solution for 45 s until it turned milky, activating the microbubbles. The activated MBs were injected retro-orbitally, enabling detailed visualization of their trajectories within a 500-µm-thick coronal brain section. The fUS device generated high-resolution ULM images, as well as velocity and flow direction intensity maps spanning the full depth of the mouse cerebrum. Imaging sessions were conducted over 1.5–2 h to ensure steady-state measurements. The ICONEUS One built-in software processed ULM, velocity, and flow direction data.

### Neurovascular coupling assessment

Neurovascular coupling was assessed using fUS imaging (ICONEUS, Iconeus One, Paris, France) to measure cerebral blood flow changes in response to neuronal stimulation. Experiments were performed on lightly anesthetized mice (0.5–1% isofluorane gas in room air) to ensure consistent physiological conditions and minimize motion artifacts. Mice were maintained at a stable body temperature of 37 °C using a heating pad, and temperature and respiration, were monitored throughout the procedure. We employed a mechanical stimulation paradigm using a cotton swab connected to a servo motor, which was controlled by an Arduino microcontroller. The Arduino was programmed to deliver precise stimulation cycles consisting of 30 s of stimulation followed by 30 s of rest, operating at a frequency of 5 Hz. This setup ensured consistent and reproducible stimulation, allowing for reliable assessment of neurovascular coupling dynamics. Each stimulation train consisted of seven pulses. Each train was repeated at least three times per side, with 5–10-min intervals between trials to avoid habituation or adaptation. Baseline CBF was recorded for 30 s prior to stimulation, and changes in CBF during and after stimulation were recorded in real time. High-resolution imaging enabled precise visualization and quantification of blood flow velocities in cortical and subcortical regions of interest. Following data acquisition, a custom MATLAB script was used to process the NVC responses. The script extracted individual NVC responses, applied baseline shift corrections to account for any drift, and averaged all the data consisting of over 40 responses per mouse. This approach ensured robust and accurate quantification of the cerebrovascular responses, enabling reliable comparisons across experimental conditions. CBF changes were expressed as a percentage increase from baseline, and the temporal dynamics of the response were analyzed to evaluate NVC. Statistical analyses were conducted using GraphPad Prism to identify significant differences in NVC parameters, with *p*-values < 0.05 considered statistically significant.

### Postprocessing and image analysis of fUS results and ULM

Postprocessing of fUS and ULM images was conducted using both the ICONEUS One fUS imager’s built-in software (ICONEUS, France) and additional analytical tools. The original data included high-resolution ULM images, vascular velocity maps, and flow directionality maps, providing comprehensive insights into the cerebrovascular dynamics.

For further analysis, ImageJ software (version 1.53t, Wayne Rasband, National Institutes of Health, USA) was utilized based on our previously established image processing protocol [18]. High-resolution ULM maps were analyzed to assess vascular coverage and microvascular density. Cortical and hippocampal regions were identified according to anatomical localization and characteristic vascular branching patterns (Fig. 1B). A supervised machine learning algorithm, implemented via the “Trainable Weka Segmentation” plugin in ImageJ, was used to classify vasculature and extravascular spaces. Binary images of segmented vascular structures were created to represent the 2D cross-sections of the 3D volume of interest (VOI), enabling precise quantification of vascular area coverage. Further analysis of segmented binary vascular masks was performed using the “Local Thickness” function to evaluate vessel diameter distribution. Vessels with diameters exceeding 20 μm were excluded to isolate the density of the microvascular network, which was defined by vessels with diameters below 20 μm.

Velocity and flow directionality maps were utilized to determine flow rates within the middle cerebral arteries branching from the Circle of Willis. RGB velocity images were converted from hexagonal to grayscale format using MATLAB R2023a. Flow rates were calculated by integrating vessel diameter and velocity data, using the following formula:

$$Q\;(\text{flow rate})=\frac{\text{Velocity}}{2}\pi {r}^{2}$$

where *r* represents the radius of the arterial branches. Arterial branches with diameters exceeding 50 μm were identified and measured using flow directionality and local diameter maps, ensuring accurate calculation of flow rates. This comprehensive analysis pipeline facilitated precise quantification of microvascular attributes and flow dynamics, providing valuable insights into PTX-induced changes in cerebrovascular function.

### Statistical analysis

Statistical analyses were performed using GraphPad Prism 10 software (La Jolla, CA, USA). Data are presented as mean ± standard error of the mean (SEM). Comparisons between experimental groups were conducted using two-tailed or paired Student’s *t*-tests, as appropriate. A minimum of three independent measurements (*n* ≥ 3) were performed for all data, with the exact number of animals used specified in the corresponding figure legends. Statistical significance was defined as *p* < 0.05, with levels of significance indicated as follows: *p < 0.05, **p < 0.01, ***p < 0.001. Data points affected by factors outside the experimental protocol, such as suboptimal cranial window quality, were excluded from the analysis to ensure accuracy and reliability of the results.

## Results

### PTX treatment reduces vascular density in the cerebral cortex and hippocampus

**Fig. 2:**
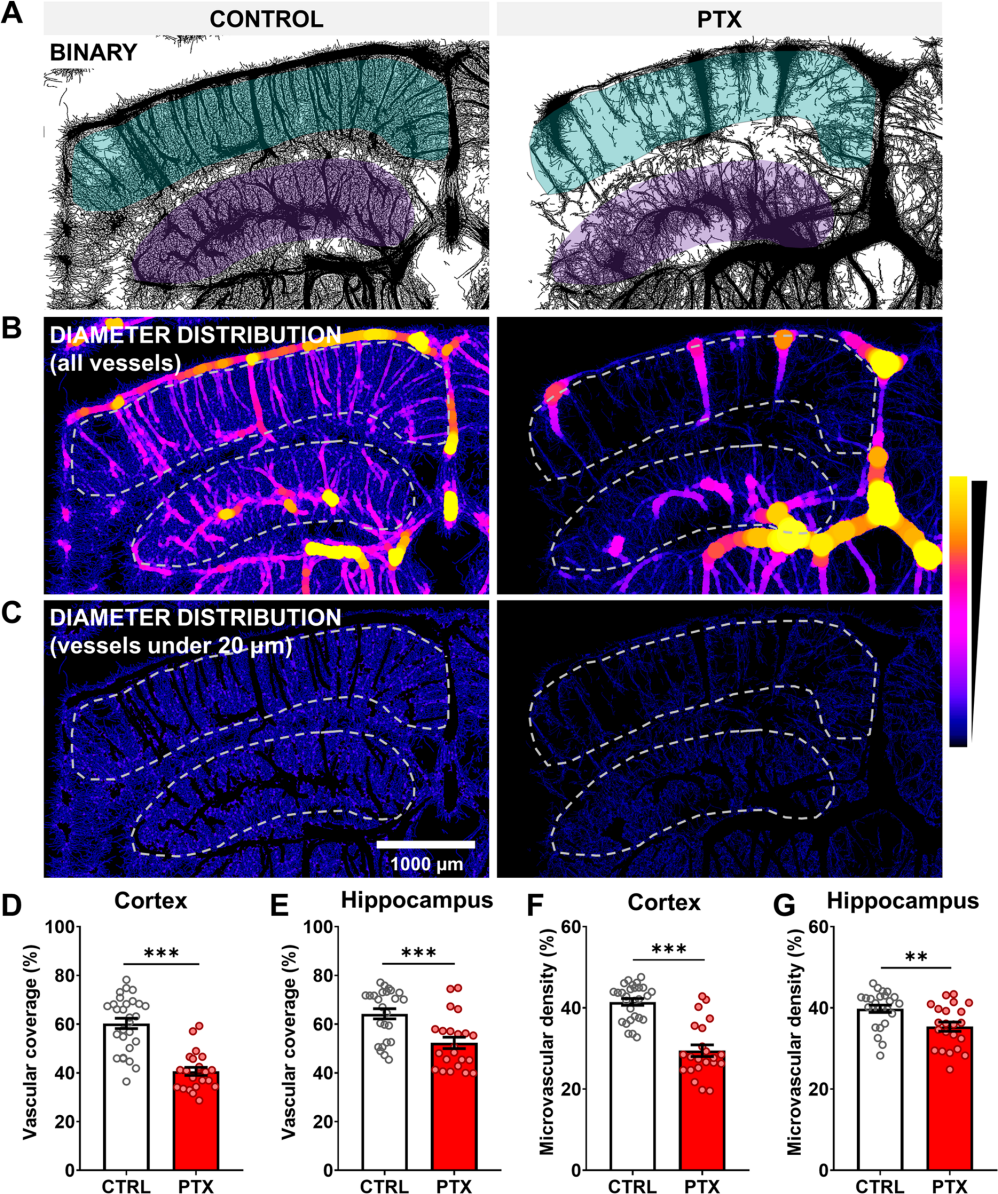
Microvascular rarefaction in the cerebral cortex and hippocampus of PTX-treated mice. **A** Segmented brain vasculature images from control and PTX-treated mice, generated using ultrasound localization microscopy (ULM) and analyzed with supervised machine learning^18^. The vascular coverage was significantly reduced in the cortex **D** and hippocampus **E** of PTX-treated mice compared to controls. **B** Vascular diameter distribution for the total vasculature in cortical and hippocampal regions, showing reduced presence of smaller vessels (capillaries and precapillary vessels) in PTX-treated mice. Quantification confirms significant differences in the vascular diameter distribution in the cortex **H** and hippocampus **I** of PTX-treated animals. **C** Masked images isolating microvasculature (< 20-μm diameter), highlighting a reduction in microvascular density in PTX-treated mice. Quantitative analysis demonstrates significant reductions in microvascular density in the cortex **F** and hippocampus **G**. All values are presented as mean ± SEM, with *n* ≥ 10. Statistical comparisons were made using two-tailed *t*-tests or paired *t*-tests for diameter distribution curves. Statistical significance: ^∗^*p* < 0.05, ^∗∗^*p* < 0.01, ^∗∗∗^*p* < 0.001

Cerebral blood flow heavily relies on the complexity of the cerebromicrovascular network. The density of microvessels is so high that nearly every neuron is closely associated with a capillary. However, during aging and in conditions associated with accelerated microvascular aging, a reduction in microvascular density and network complexity is often observed [25–27]. In this study, PTX treatment led to a sustained reduction in cerebromicrovascular density within the cerebral cortex and hippocampus (Fig. 2), two brain regions essential for higher cognitive functions, including learning and memory. Further analysis of vascular diameter distribution revealed notable differences, particularly in the cortical areas, where the proportion of the smallest microvessels decreased significantly. To specifically assess the density of the smallest cerebral vessels, larger vessels (> 20 μm in diameter) were excluded from the analysis. This revealed a significant reduction in the density of smaller arterioles, capillaries, and venules in both the cerebral cortex (Fig. 2F) and hippocampus (Fig. 2G), indicating a loss of microvascular complexity in PTX-treated animals.

### PTX treatment affects CBF

**Fig. 3:**
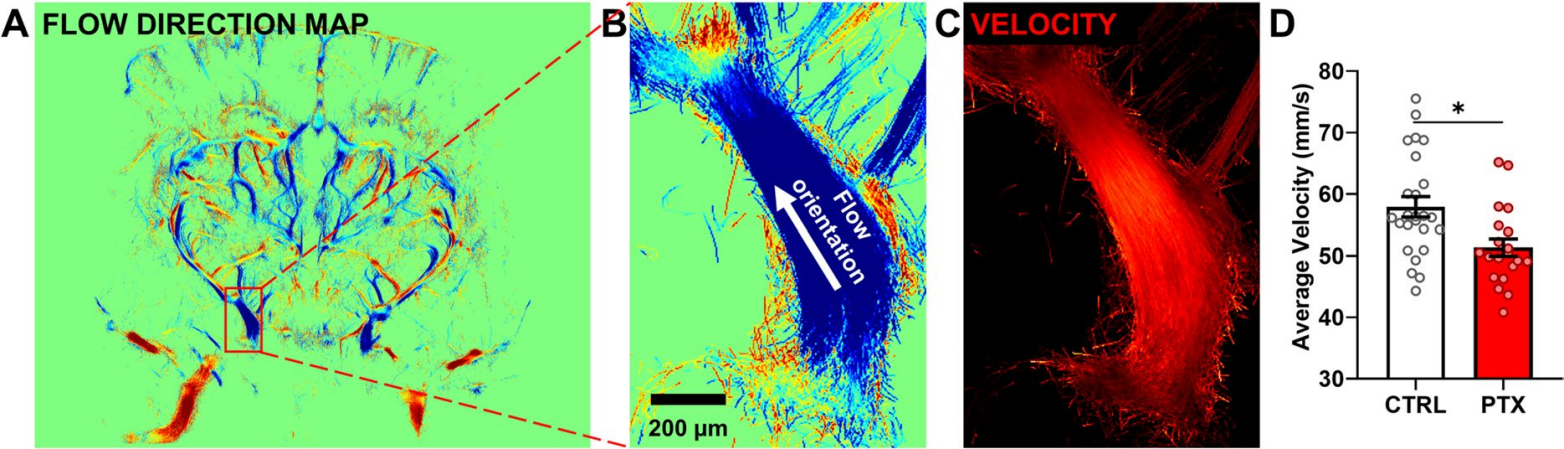
Effects of PTX treatment on blood velocity and flow in the middle cerebral artery. **A**, **B** Representative flow directionality maps of brain vasculature, showing the flow direction from the Circle of Willis toward the brain, indicated by blue-colored regions. **C** Blood velocity map of the middle cerebral artery. **D **Quantification of the average blood velocity in the middle cerebral artery, showing a significant reduction in PTX-treated animals compared to controls. All values are presented as mean ± SEM, with *n* ≥ 10. Groups were compared using Student’s *t*-test, with statistical significance set at *p* < 0.05

Blood flow in the middle cerebral arteries is a key determinant of baseline brain perfusion. The brain’s primary arterial supply system converges at the Circle of Willis, from which critical branches, including the middle cerebral arteries, originate (Fig. 3A). The anatomy of the mouse cerebrovascular network and its blood flow orientation are illustrated in Fig. 3A and B. In PTX-treated animals, the cumulative average blood velocity in the middle cerebral arteries decreased significantly (Fig. 3C, D), consistent with the idea that development of chemobrain is associated with impaired brain perfusion.

### PTX treatment impairs cortical NVC responses

**Fig. 4:**
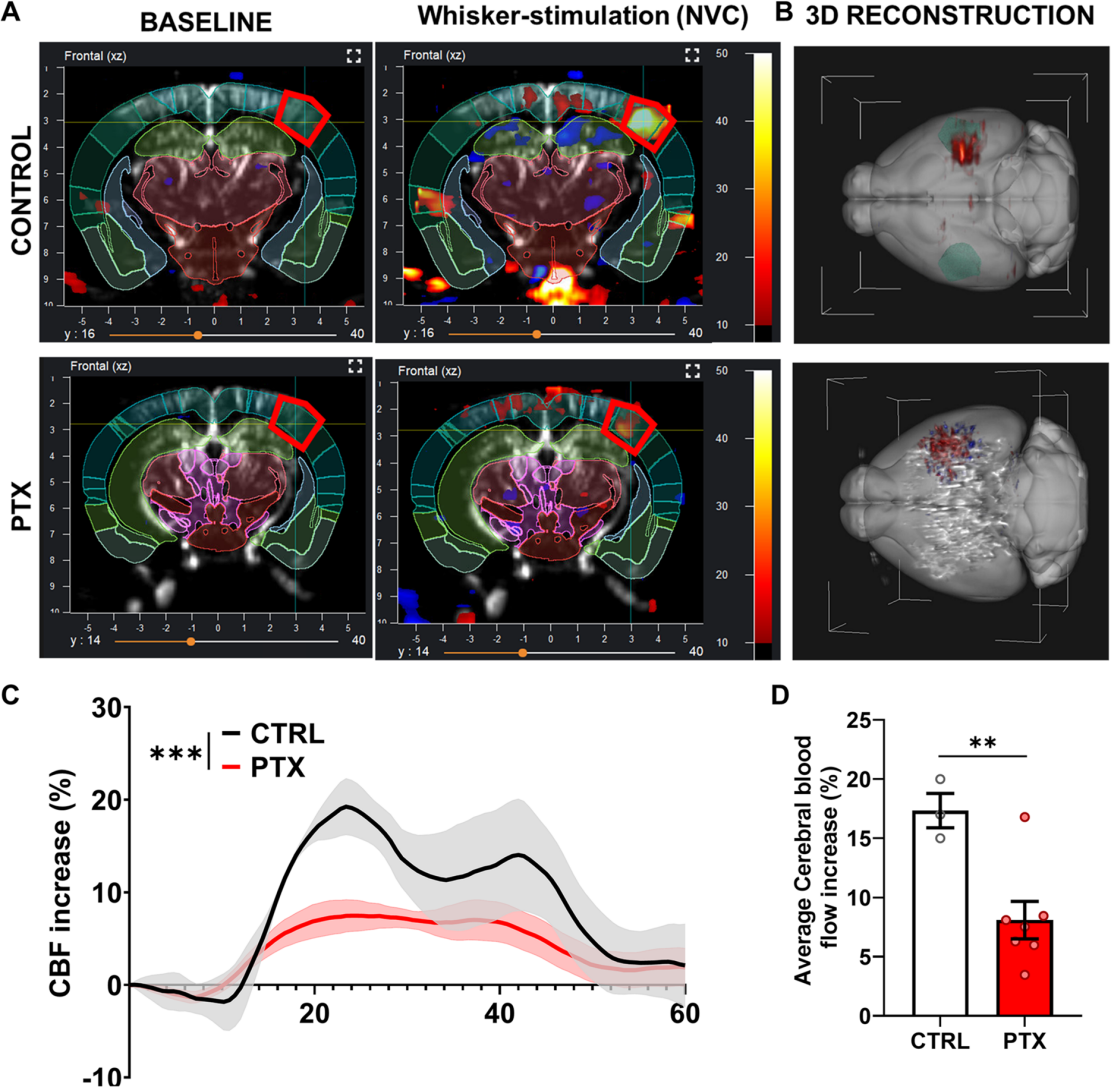
Impairment of neurovascular coupling (NVC) in PTX-treated mice. **A** Representative functional ultrasound (fUS) images showing cerebral blood flow (CBF) changes in response to whisker stimulation in control and PTX-treated mice. The regions of interest (barrel cortex) are highlighted in red. **B** Three-dimensional reconstruction of NVC responses in the barrel cortex of control and PTX-treated mice, demonstrating reduced CBF activation in PTX-treated animals. **C** Time-course analysis of CBF changes in response to contralateral whisker stimulation, showing significantly diminished NVC responses in PTX-treated mice compared to controls. **D** Quantification of the average CBF increase in the barrel cortex, confirming a significant reduction in NVC responses in PTX-treated mice. All values are presented as mean ± SEM, with *N* ≥ 3. Statistical comparisons were performed using two-tailed *t*-tests or paired *t*-tests for time-dependent curves. Statistical significance: ^∗^*p* < 0.05, ^∗∗^*p* < 0.01, ^∗∗∗^*p* < 0.001

The ICONEUS fUS system, combining ULM and vascular velocity measurements, enables real-time assessment of relative blood flow fluctuations [18]. This system was used to evaluate NVC responses in the somatosensory barrel cortex evoked by contralateral whisker stimulation (Fig. 4A). This approach monitors blood flow changes throughout the entire brain in three dimensions, providing precise reconstructions of affected areas (Fig. 4B). Whisker stimulation in control animals elicited a robust NVC response localized to the barrel cortex (Fig. 4B). In PTX-treated animals, however, the amplitude of cerebral blood flow changes was markedly smaller compared to controls (Fig. 4C). Quantitative analysis of the average increase in CBF confirmed a significantly reduced NVC response in PTX-treated animals, reflecting a partial impairment in neurovascular function.

## Discussion

This study builds on our previous findings, demonstrating that PTX treatment induces persistent microvascular dysfunction that likely underlies CICI [15]. Specifically, we observed long-lasting reductions in cerebromicrovascular density, impaired NVC responses, and altered CBF. These findings further support the critical role of cerebromicrovascular health in CICI and provide new insights into the long-term vascular effects of PTX on the brain.

In our earlier work, we identified PTX-induced endothelial senescence as a key mechanism driving microvascular impairment and BBB disruption [15]. These pathophysiological changes were causally linked to cognitive deficits in PTX-treated mice and could be reversed by senolytic treatments [15]. The current study confirms the long-term impact of PTX on structural and functional integrity of the cerebromicrovascular network while revealing additional insights into its effects on CBF regulation.

Our findings of reduced microvascular density in the cerebral cortex and hippocampus are consistent with our earlier observations using two-photon microscopy and optical coherence tomography [15]. These regions are critical for learning and memory, and their vascular rarefaction is associated with impaired metabolic support for neural signaling. Notably, microvascular rarefaction is also a hallmark of the aging brain and has been causally linked to cognitive impairment, further emphasizing the importance of maintaining the integrity of the cerebromicrovascular network for cognitive health [25,26,28]. Importantly, our previous study demonstrated that the elimination of senescent endothelial cells restored capillary density, underscoring the therapeutic potential of targeting vascular senescence [15].

The current study revealed significant reductions in basal blood flow in the middle cerebral arteries, consistent with an increased cerebrovascular resistance due to PTX-induced microvascular rarefaction and endothelial dysfunction [15]. We further characterized the impact of PTX on NVC responses using functional ultrasound imaging, to assess CBF changes elicited by whisker stimulation. The observed reduction in NVC amplitude aligns with our previous findings that impaired bioavailability of endothelial-derived nitric oxide is critical for impaired functional hyperemia [15]. These results suggest that PTX-induced endothelial dysfunction disrupts NVC, contributing to the neuronal dysfunction observed in CICI. Similarly, impaired NVC is also a well-documented feature of aging and has been directly associated with cognitive decline in both animal models [29–33] and humans [34–38]. Our earlier work demonstrated that senolytic treatments in PTX-treated mice restored NO-mediated NVC responses, highlighting their potential in mitigating PTX-induced vascular dysfunction and supporting their role in preserving neurovascular health [15]. We propose that the reduced capillary support in cortical and hippocampal regions likely limits perfusion under conditions of increased metabolic demand, exacerbating cognitive deficits.

Together, these findings highlight the importance of preserving microvascular health in mitigating CICI. Our previous work demonstrated the efficacy of senolytic treatments in restoring BBB integrity, reducing neuroinflammation, and improving cognitive performance in PTX-treated mice [15]. However, translating these findings into clinical applications requires identifying low-toxicity, well-tolerated, and effective therapeutic regimens that can be safely administered to cancer patients without interfering with chemotherapy efficacy. Future studies should explore the translational potential of these therapies in clinical settings, particularly in patients at high risk for CICI, while prioritizing strategies that minimize adverse effects and enhance long-term cerebrovascular protection.

This study focused on vascular outcomes and did not directly assess endothelial senescence, which we previously identified as a key driver of microvascular dysfunction [15]. Future studies should integrate molecular analyses to confirm the persistence of endothelial senescence in various brain regions. Additionally, investigating sex-specific differences and the potential impact of PTX on other brain cell types, including pericytes, could further elucidate the mechanisms of CICI.

While our findings strongly suggest that PTX-induced cerebrovascular dysfunction contributes to CICI, this study did not include direct cognitive testing. However, our previous work [15] demonstrated that cerebrovascular impairment is causally linked to cognitive deficits in PTX-treated mice. Future studies should integrate behavioral and cognitive assessments alongside advanced cerebrovascular imaging to establish a direct functional correlation between vascular dysfunction and cognitive impairment.

Moreover, fUS imaging provides a powerful tool for non-invasive, high-resolution cerebrovascular assessment. Expanding this approach to investigate the effects of other chemotherapeutic agents on the neurovascular system would help determine whether cerebrovascular dysfunction is a common mechanism underlying CICI across different chemotherapy regimens [39–41]. Additionally, using fUS to evaluate potential therapeutic interventions aimed at enhancing cerebrovascular health may offer promising strategies to mitigate CICI and improve long-term cognitive outcomes in cancer survivors.

It should also be acknowledged that we used non-tumor-bearing mice to isolate the direct effects of PTX on cerebrovascular function. While this approach minimizes confounding factors related to tumor biology, it does not fully recapitulate the clinical scenario in which chemotherapy is administered alongside tumor progression and systemic inflammatory changes. Tumor-secreted cytokines and metabolic alterations have been implicated in cancer-related cognitive impairment, independent of chemotherapy. Future studies could compare PTX-treated tumor-bearing mice with non-tumor-bearing models to disentangle the relative contributions of chemotherapy-induced vascular dysfunction and tumor-related systemic effects on cognitive impairment.

In conclusion, this study reinforces the critical role of microvascular dysfunction in CICI, demonstrating that PTX induces long-lasting impairments in microvascular density, NVC responses, and CBF. By contextualizing these findings within our previous work, we provide a comprehensive framework for understanding the cerebrovascular contributions to CICI and highlight the therapeutic potential of targeting vascular health to preserve cognitive function in cancer survivors.

## Abbreviations

BBB: Blood-brain barrier
CBF: Cerebral blood flow
CICI: Chemotherapy-induced cognitive impairment
fUS: Functional ultrasound imaging
MBs: Microbubbles
NVC: Neurovascular coupling
PTX: Paclitaxel
TPX: Polymethylpentene
ULM: Ultrasound localization microscopy
